# Metagenomic Bioprospecting of Bioremediation‐Related Genes in Amazonian Aquatic Ecosystems

**DOI:** 10.1111/1758-2229.70435

**Published:** 2026-10-21

**Authors:** Thais Mayara Castro dos Anjos, Daralyns Borges Macedo, Victor Benedito Costa Ferreira, Carlos Willian Dias Dantas, Edian Franklin Franco de Los Santos, Oscar Victor Cardenas‐Alegria, Rommel Thiago Jucá Ramos

**Affiliations:** ^1^ Laboratory of Bioinformatics and Genomics of Microorganisms Institute of Biological Sciences, Federal University of Pará Belém Brazil; ^2^ Laboratory of Simulation and Computational Biology, High Performance Computing Center Federal University of Pará Belém Brazil; ^3^ Santo Domingo Institute of Technology Santo Domingo Dominican Republic

**Keywords:** copper, freshwater, heavy metals, mercury, metagenomics, microbial resistance

## Abstract

Environmental monitoring of contaminated freshwater systems still lacks curated genomic resources tailored to bioremediation‐oriented screening. In this study, we developed a curated catalogue of 546 genes from 80 microbial species associated with heavy‐metal resistance, hydrocarbon degradation, detoxification and related functions and applied it to river metagenomes from four municipalities in Pará, Brazilian Amazon: Altamira, Bragança, Marabá and Paragominas. Functional annotation was performed using eggNOG‐mapper and metagenomic reads were aligned against the curated catalogue with Bowtie2. Mercury‐ and copper‐related genes were the most frequently represented functional categories across all river systems, a pattern that may reflect both sequence representation in the metagenomes and the unequal composition of the curated reference catalogue. Bragança showed the largest detected gene repertoire, whereas diversity, Jaccard, UpSet and Dunn‐based analyses revealed marked spatial heterogeneity and sites of localized functional enrichment. Recurrently associated bacterial taxa included *Acinetobacter*, *Enterobacter*, *Pseudomonas*, *Stenotrophomonas*, *Cupriavidus* and *Sphingopyxis*. These findings indicate that the catalogue provides a targeted genomic reference for environmental screening and bioprospecting, although metagenomic detection represents functional potential rather than experimentally confirmed remediation activity.

## Graphical abstract

Overview of the study workflow and main findings. Metagenomes from Amazonian rivers in Pará, Brazil (Altamira, Bragança, Marabá and Paragominas) were processed through DNA extraction and quality control, followed by bioinformatic read processing and functional annotation to build a curated catalogue of 546 bioremediation genes from 80 microbial species. Mercury (Hg) and copper (Cu) resistance genes dominated across sites, with Bragança showing the highest richness and the resulting resource enables molecular surveillance of polluted waters and supports biotechnological strategies for remediation and sustainable management of Amazonian freshwater ecosystems.

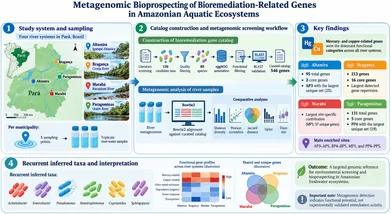

## 1 Introduction

Freshwater ecosystems are increasingly affected by hydrocarbons, heavy metals, pesticides and other persistent pollutants derived from urbanization, mining, agriculture and industrial activities. These contaminants can disrupt aquatic communities and compromise water quality, highlighting the need for sustainable remediation strategies. Bioremediation uses the metabolic and adaptive capacities of microorganisms to transform, immobilize, or detoxify pollutants and genomic approaches have expanded the ability to identify the genes and pathways underlying these functions. However, despite the availability of general genomic databases, targeted and curated resources integrating microbial taxa, functional annotations and pollutant‐related genes remain limited, particularly for tropical and biodiversity‐rich regions such as the Amazon (Bala et al. 2022; Datta et al. 2020).

The Amazon basin is increasingly exposed to anthropogenic pressures associated with urban expansion, deforestation, agriculture, mining and hydroelectric development, yet its aquatic microbial communities remain insufficiently explored as sources of bioremediation‐related functions (Lapola et al. 2023; Carvalho et al. 2020). To address this gap, we developed a curated catalogue of genes associated with pollutant resistance, degradation and detoxification and applied it to metagenomic datasets from river systems in Altamira, Bragança, Marabá and Paragominas, Pará, Brazil. We hypothesized that differences in local anthropogenic pressure would be associated with spatial variation in the distribution of these functions. Accordingly, this study aimed to identify shared and site‐specific bioremediation‐related patterns and to provide a targeted genomic resource for environmental screening and bioprospecting in Amazonian aquatic ecosystems.

## 2 Materials and Methods

### 2.1 Genetic Heritage Authorizations and Data

All selected sampling points were previously authorized and registered under Sistema de Autorização e Informação em Biodiversidade (SISBIO), under protocol number 81367‐2. Additionally, the sampling was recorded in Sistema Nacional de Gestão do Patrimônio Genético (SISGEN) under registration number AD70806.

### 2.2 Cities and Sample Collections

This study was conducted in the municipalities of Altamira, Bragança, Marabá and Paragominas, located in the state of Pará, Brazil, where samples were collected in Igarapé Altamira, Cereja, Itacaiúnas and Uraim rivers, respectively.

Samples were collected from five points per city, with triplicate samples taken at each location. The selection of sampling sites was based on the anticipated levels of water contamination. The first sampling point corresponded to the least impacted area, typically located near the source of the water body, while the subsequent points were positioned progressively downstream, where contamination levels were expected to increase. Consequently, Point 5 was designated as the site most likely to exhibit the highest level of contamination.

Because sampling sites were selected according to expected contamination gradients, the study design should be interpreted as a targeted ecological screening rather than a random spatial survey. Consequently, the possibility of selection bias toward potentially impacted sectors was considered when interpreting spatial patterns. The geographic distribution of the five sampling points in Altamira, Bragança, Marabá and Paragominas, located along the Igarapé Altamira, Cereja, Itacaiúnas and Uraim rivers, respectively, is shown in Figure 1A–D.

**FIGURE 1:**
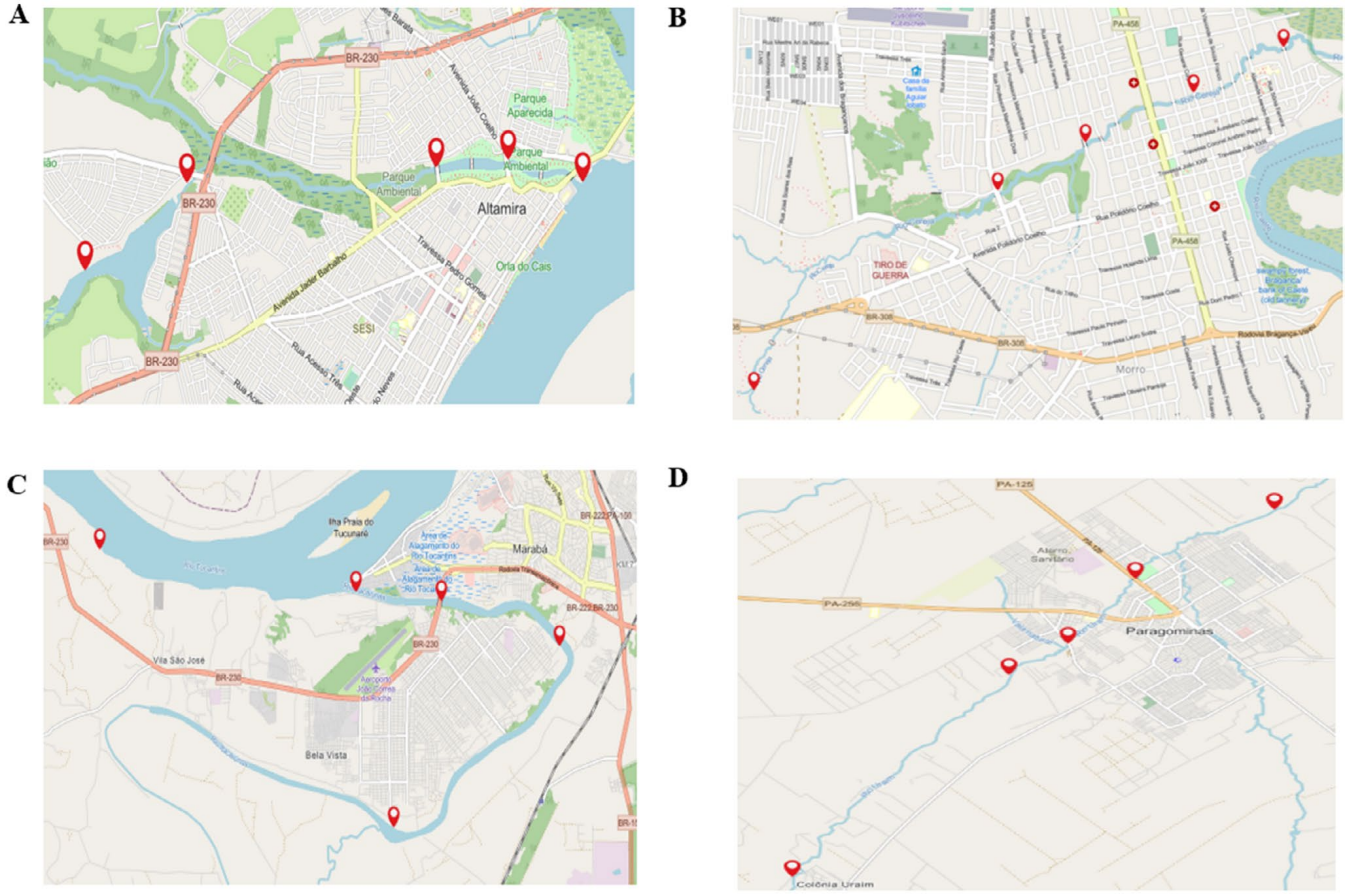
Geographic location of the sampling sites in the municipalities of Altamira (A), Bragança (B), Marabá (C) and Paragominas (D), Pará State, Brazilian Amazon. Red markers represent the river water sampling points used for metagenomic screening of bioremediation‐related genes. Maps were produced in ArcGIS Pro (Esri) using OpenStreetMap basemap data (OpenStreetMap contributors).

### 2.3 Metagenomic DNA Collection and Processing

The water collection for metagenomic analysis was performed using a 5‐l Van Dorn bottle, which was disinfected and washed with distilled water prior to sampling. The collected material was then transferred into sterile polypropylene bottles of the same capacity, with one bottle used per replicate. Before and after each collection, the Van Dorn bottle was disinfected with a 10% sodium hypochlorite solution, followed by three washes with distilled water. The sample bottles were placed on ice and transported to the laboratory.

The collected water underwent pre‐filtration before being filtered under negative pressure through a nitrocellulose membrane with pore sizes of 14 μm (Qually) and 0.22 μm (Millipore), respectively. The 0.22 μm filtered membranes were transferred to 15 mL polypropylene tubes (Falcon tubes) containing a DNA preservation solution (50 mM Tris–HCl, 500 mM NaCl, 125 mM EDTA, pH 8.0) and stored at −80°C for subsequent metagenomic deoxyribonucleic acid (DNA) extraction.

Metagenomic DNA was extracted using the DNeasy PowerSoil Pro Kit (QIAGEN), following the manufacturer's protocol. DNA concentration was measured by spectrophotometry at 260 and 280 nm using a NanoDrop 1000 spectrophotometer. To be considered suitable for sequencing, DNA samples needed to reach a minimum concentration of 50 ng/μL, with a purity index (PI) between 1.4 and 2.0. To further evaluate the quality of the extracted DNA, purity indices (PI) were determined based on the A260/A280 absorbance ratio, with acceptable values ranging between 1.8 and 2.0. DNA samples meeting the expected PI criteria were stored at −20°C until further use.

### 2.4 Sequencing and Bioinformatics Analysis

#### 2.4.1 Sequencing and Quality Assessment

Metagenomic DNA was sequenced on a NovaSeq 6000 platform using 150‐bp reads. Raw sequencing data in FASTQ format were initially assessed with FastQC (Andrews 2010) to evaluate read quality, length distribution, adapter content and other sequencing characteristics. Quality filtering and trimming were subsequently performed using Trimmomatic (Bolger et al. 2014). Reads shorter than 75 bp and regions with an average Phred score below 20 were removed, together with any residual adapter sequences.

#### 2.4.2 Assembly and Functional Analysis of Contigs

During the assembly stage, the MEGAHIT program (Li et al. 2015) was used to assemble the reads into contigs, applying the “meta‐large” parameter to optimize the assembly of complex metagenomic datasets. The assembled contigs were then evaluated for quality using MetaQuast (Mikheenko et al. 2016).

For functional analysis, the contigs were processed using Prokka v.1.14.5 (Seemann 2014) and the GOFEAT web platform (Araujo et al. 2018). The curated catalogue developed in this study was used to identify bioremediation‐related genes associated with heavy‐metal resistance, pollutant degradation, detoxification, biofilm formation and related functions.

### 2.5 Acquisition and Analysis of Sequences Related to Bioremediation

#### 2.5.1 Structured Literature Screening and Catalogue Construction

A structured literature‐guided screening, rather than a formal systematic review, was conducted using Google Scholar, PubMed, Consensus, ScienceDirect and Scopus to identify bacterial species associated with bioremediation, pollutant resistance, hydrocarbon degradation and environmental detoxification. Studies published between 2010 and 2024 were screened according to their relevance to bioremediation, species‐level taxonomic resolution, genome availability and compatibility with downstream functional annotation. Duplicate records were removed manually and no quantitative quality‐scoring system was applied.

Candidate genomes were retrieved from National Center for Biotechnology Information (NCBI), with RefSeq assemblies prioritized whenever available. Records lacking species‐level identification, sufficient genomic information, adequate annotation, or compatibility with the functional‐screening workflow were excluded. After this filtering step, the initial set of 130 candidate taxa was reduced to 80 species suitable for catalogue construction.

The selected genomes were functionally annotated using eggNOG‐mapper (Cantalapiedra et al. 2021) in DIAMOND search mode. Orthology assignments were based on the eggNOG v5 database using the BLOSUM62 scoring matrix, while the default E‐value threshold for seed‐ortholog identification was retained to maintain consistency with the standard annotation workflow. Sequences associated with resistance, degradation, detoxification and related functions were subsequently selected and validated by Basic Local Alignment Search Tool (BLAST). Only matches showing at least 95% similarity to the expected targets were retained to reduce ambiguous assignments. This process resulted in a final curated catalogue of 546 bioremediation‐related genes, as summarized in Figure 2.

**FIGURE 2:**
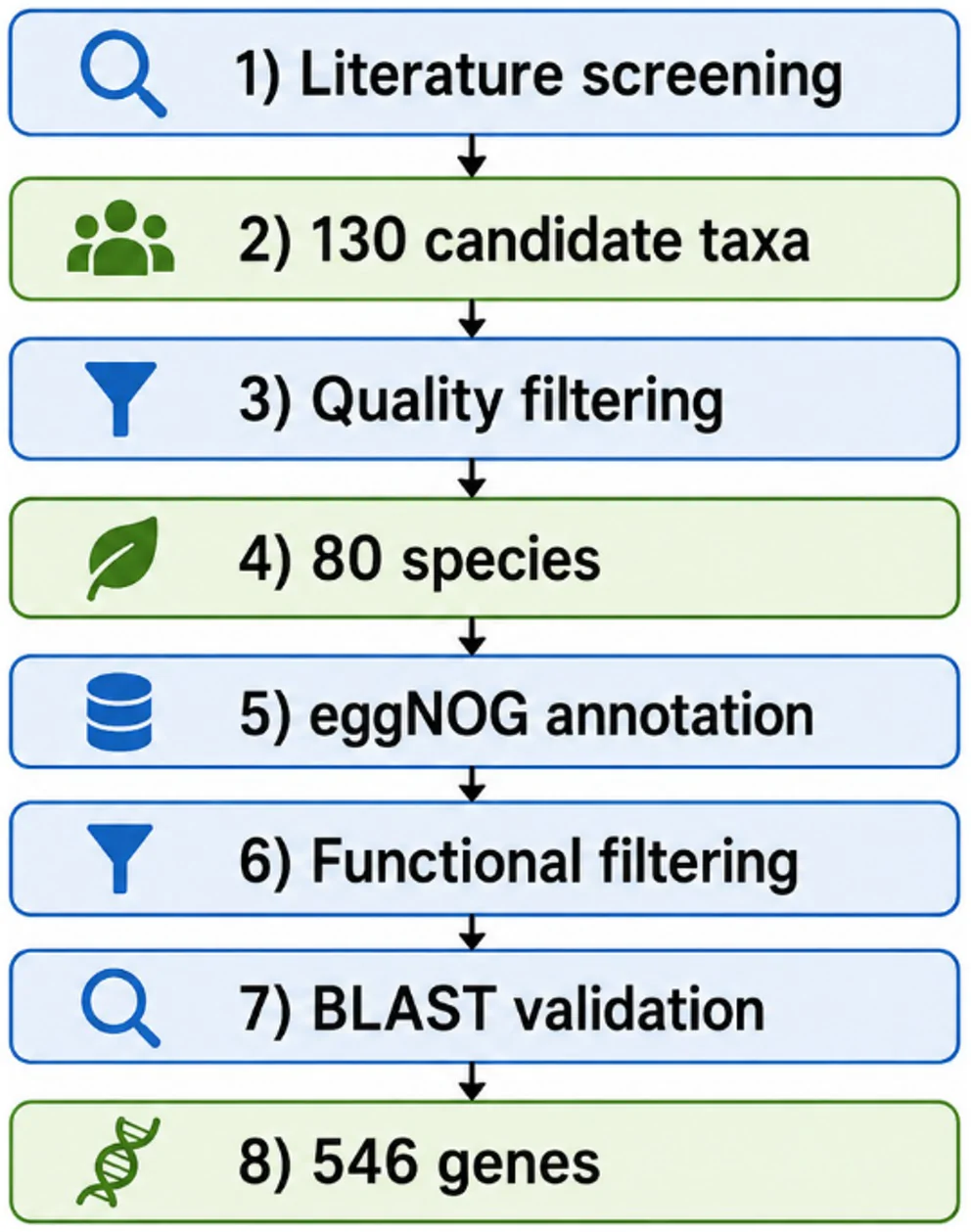
Workflow used for the construction of the curated bioremediation gene catalogue. The process comprised structured literature screening, identification of 130 candidate taxa, quality filtering, selection of 80 species, functional annotation with eggNOG‐mapper, functional filtering, BLAST validation and retention of a final set of 546 genes.

#### 2.5.2 Database Processing

The curated genomic database was indexed using Bowtie2 (Langmead and Salzberg 2012) and used as the reference for read alignment. Paired‐end metagenomic reads were aligned against the custom database index using the ‐x option, with forward and reverse reads provided through the −1 and −2 parameters, respectively. Sequence Alignment/Map (SAM) output files were generated using the ‐S option. Alignment was performed allowing up to one mismatch in the seed region (‐N 1) and using 10 processing threads (−p 10). All other Bowtie2 parameters were kept at default settings. Because the screening was performed against a curated functional reference, alignment results were interpreted as evidence of sequence association with catalogue entries rather than direct proof of gene activity. Reads aligning to homologous targets were therefore treated conservatively in downstream summaries, prioritizing robust detection patterns over fine‐scale assignment of ambiguous matches.

Under the default Bowtie2 reporting mode, one primary alignment was reported for each read or read pair. When multiple valid alignments were available, the reported alignment represented the best alignment identified by the search procedure; equally scoring alternatives were not counted separately. Consequently, gene‐ and taxon‐level assignments involving highly homologous reference sequences were interpreted cautiously.

Alignment results were generated in SAM format and subsequently converted to Binary Alignment/Map (BAM) format using SAMtools (Li et al. 2009). BAM files were indexed with samtools index and reference‐level read counts were obtained separately for each biological replicate using samtools idxstats, which reports the reference identifier, reference length and numbers of mapped and unmapped reads for each catalogue entry. To account for differences in sequencing depth, gene counts were normalized as counts per million mapped reads (CPM) within each replicate. For site‐level heatmap visualization, normalized counts from the three biological replicates were averaged for each sampling point and transformed into gene‐wise z‐scores across sites. Replicates were retained separately for Jaccard analysis, in which a gene was considered present when at least one mapped read was detected in the corresponding replicate. For site‐level presence–absence summaries and UpSet analyses, a gene was classified as present only when detected in at least two of the three biological replicates, thereby reducing the influence of isolated detections.

Downstream processing, visualization and exploratory analyses were performed in R. Data manipulation and formatting were carried out using the dplyr and reshape2 packages. Heatmaps of gene abundance were generated with pheatmap, correlation matrices were visualized using ggcorrplot and corrplot, and bar plots and complementary graphics were produced with ggplot2. Ecological analyses, including diversity metrics and compositional similarity, were conducted with the vegan package, while intersections of shared and site‐specific genes were visualized using UpSetR. Together, these packages supported the organization of alignment‐derived count tables, the generation of summary spreadsheets, and the graphical representation of pollutant distribution, microbial profiles and gene overlap across sampling sites. Because the analyses were based on metagenomic read alignment, gene abundance, and presence–absence data, the detected patterns were interpreted as sequence representation rather than gene expression or functional activation. Accordingly, terms such as ‘gene abundance’, ‘gene detection’, ‘functional profile’ and ‘sequence association’ were used throughout the manuscript, since transcriptomic data were not generated in this study.

Taxonomic associations were derived from the host‐species metadata linked to each reference sequence in the curated catalogue. Therefore, the reported taxa represent the source organisms associated with matched catalogue entries and do not constitute an independent taxonomic classification of the metagenomic reads.

### 2.6 Statistical and Visual Analysis of Genomic Data

Pearson correlation, Shannon diversity, Jaccard dissimilarity and Dunn's post hoc test were used to address distinct but complementary biological questions. Pearson correlation was applied to metagenomic gene‐abundance profiles to assess similarity in the relative distribution of bioremediation‐related sequences among sampling sites. The Shannon index was calculated from gene‐abundance data to characterize within‐site richness and evenness. Jaccard dissimilarity was calculated from a binary presence–absence matrix using the vegdist() function of the vegan package with method = ‘jaccard’, allowing compositional turnover to be assessed independently of abundance magnitude.

Data normality was evaluated using the Shapiro–Wilk test. Because gene abundance did not meet the assumptions of parametric testing, Kruskal–Wallis was applied, followed by Dunn's post hoc comparisons using dunn.test::dunn.test(). *p*‐values from the Kruskal–Wallis tests were adjusted using the Benjamini–Hochberg procedure, whereas Dunn's pairwise comparisons were adjusted using the Bonferroni correction. Adjusted *p*‐values below 0.05 were considered statistically significant. Together, these analyses described complementary dimensions of the dataset: similarity in abundance patterns, within‐site diversity, presence–absence turnover and site‐specific pairwise differentiation. None of these analyses was interpreted as evidence of gene expression, contaminant concentration, or functional activation.

## 3 Results and Discussion

### 3.1 Curated Bioremediation Gene Catalogue

A structured literature‐guided curation and genome‐screening workflow resulted in a catalogue of 546 bioremediation‐related genes derived from 80 microbial species. Although 130 candidate taxa were initially identified, only 80 species met the criteria for species‐level taxonomic resolution, genome availability, annotation quality and compatibility with downstream functional screening. The main reasons for exclusion were incomplete genomic resources, insufficient annotation for reliable processing with eggNOG‐mapper, and literature records limited to genus‐level identification. Among the retained taxa, *Bacillus brevis* , *Lysinibacillus sphaericus* and *Sphingopyxis macrogoltabida* were the most frequently represented species.

The final catalogue encompassed 15 functional categories related to resistance, degradation, detoxification, metal transport and biofilm formation, but these functions were unevenly represented. Mercury‐related genes were the predominant category, comprising 225 entries (41.21%), followed by copper‐related genes with 115 entries (21.06%) and tellurium/tellurite‐related genes with 56 entries (10.26%). Mercury‐ and copper‐associated functions therefore accounted for 62.27% of the catalogue. Other categories, including biofilm formation, arsenic resistance, general heavy‐metal responses, organic hydroperoxide resistance, chromium/chromate resistance, cadmium resistance and organic‐pollutant degradation, occurred at considerably lower frequencies. Thus, the catalogue represents a broad functional repertoire, but with a marked enrichment of mercury‐ and copper‐related functions.

The curated resource integrates sequence identity, host species, protein family, functional annotation and pollutant‐related category within a single framework. Candidate entries were filtered using pollutant‐related functional terms and validated by BLAST, retaining only matches with at least 95% similarity to the expected targets. This structure supports the screening of shared and site‐specific resistance and detoxification functions in Amazonian River metagenomes. However, the unequal representation of functional categories must be considered during downstream interpretation, because categories containing more reference sequences have greater opportunities for read assignment and may consequently appear more frequent or widespread. Therefore, the predominance of mercury‐ and copper‐related sequences in the river datasets may reflect both biological patterns in the sampled metagenomes and the composition of the curated reference catalogue.

### 3.2 Spatial Patterns of Bioremediation Genes Along the Four Rivers

Heatmaps summarizing the abundance of bioremediation‐related genes across the five sampling points in each municipality revealed marked spatial structuring along the four river systems (Altamira, Bragança, Marabá and Paragominas; Figures 3A,B, 4A,B). In total, 95, 213, 111 and 131 resistance genes were detected in Altamira, Bragança, Marabá and Paragominas, respectively, associated with 36–47 bacterial species per river.

**FIGURE 3:**
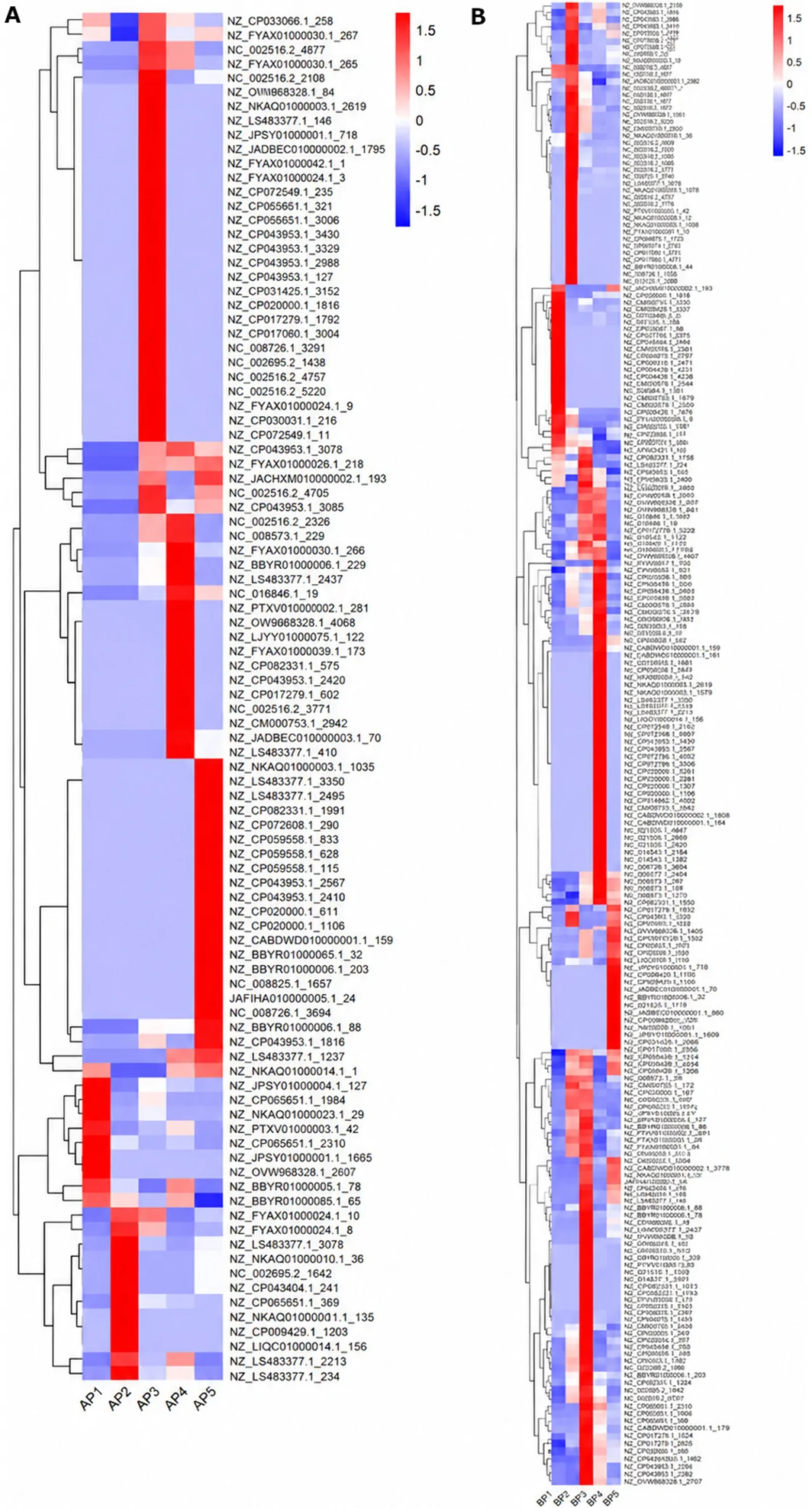
Heatmaps of bioremediation‐related gene abundance in Altamira (A) and Bragança (B). Each panel shows the relative abundance of bioremediation genes across five sampling points along the river transects (AP1–AP5 in Altamira and BP1–BP5 in Bragança), arranged from upstream to downstream. Genes are grouped according to their functional annotation and clustered by hierarchical clustering based on their abundance profiles across sites. Colours represent normalized gene abundance (*z*‐score), with warmer tones indicating higher relative abundance and cooler tones indicating lower relative abundance. Dendrograms summarize similarity patterns among genes and sampling sites. High‐resolution versions of these heatmaps, including full gene labels, are provided in Figures S1 and S2.

**FIGURE 4:**
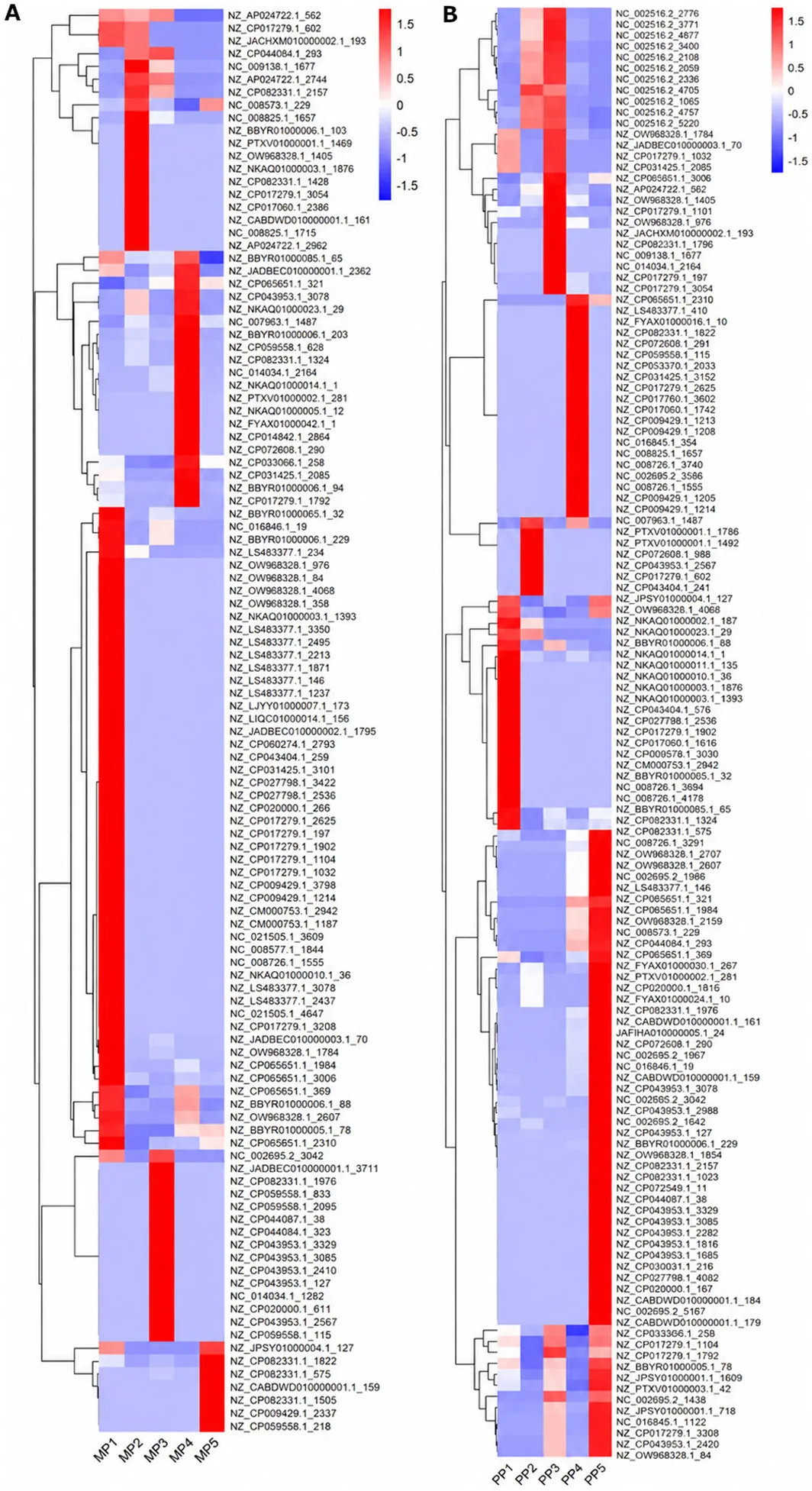
Heatmaps of bioremediation‐related gene abundance in Marabá (A) and Paragominas (B). Each panel shows the relative abundance of bioremediation genes across five sampling points along the Itacaiúnas River (MP1–MP5) and Uraim River (PP1–PP5), arranged from upstream to downstream. Genes are grouped by function and clustered by hierarchical clustering according to their abundance profiles across sites. Colours represent normalized gene abundance (*z*‐score), with warmer tones indicating higher relative abundance and cooler tones indicating lower relative abundance. Dendrograms summarize similarity patterns among genes and sampling sites. High‐resolution versions of these heatmaps, including full gene labels, are provided in Figures S3 and S4.

Heatmaps of bioremediation‐related gene abundance revealed spatial variation across the four river systems. In Altamira, higher relative abundance was concentrated at AP3–AP5, whereas AP1 and AP2 showed lower values for most genes. In Bragança, BP2 and BP4 displayed the most enriched profiles. In Marabá, MP1 showed the most distinct abundance pattern, while MP2–MP4 exhibited comparatively lower relative abundance across several genes. In Paragominas, the strongest relative enrichment was observed at PP5. Nevertheless, bioremediation‐related genes were also detected at upstream and intermediate sites, indicating that these functions were distributed throughout the sampled river sections, although their relative abundance varied among sites.

The concentration of higher relative abundance at particular sampling sites suggests localized spatial structuring of the detected functional profiles. These differences may be associated with variation in environmental conditions and anthropogenic pressures along the river courses. However, because contaminant concentrations were not directly measured, the observed patterns should be interpreted as spatial associations in metagenomic gene abundance rather than as direct evidence of contaminant‐driven selection.

### 3.3 Distribution of Contaminant‐Related Gene Categories

The distribution of bioremediation‐related genes among contaminant‐related categories showed a consistent pattern across the four municipalities (Figure 5A–D). Mercury‐related genes were the most frequently detected category in Altamira, Bragança, Marabá and Paragominas, followed by copper‐related genes. In Altamira, approximately 50 genes were associated with mercury and 25 with copper. Mercury‐related genes also exceeded 90 occurrences in Bragança, 50 in Marabá and 60 in Paragominas. Genes associated with arsenic, cadmium, chromium/chromate, tellurium/tellurite, phenol, organic hydroperoxides, biofilm formation and other pollutant‐related functions occurred at lower frequencies and varied among municipalities.

**FIGURE 5:**
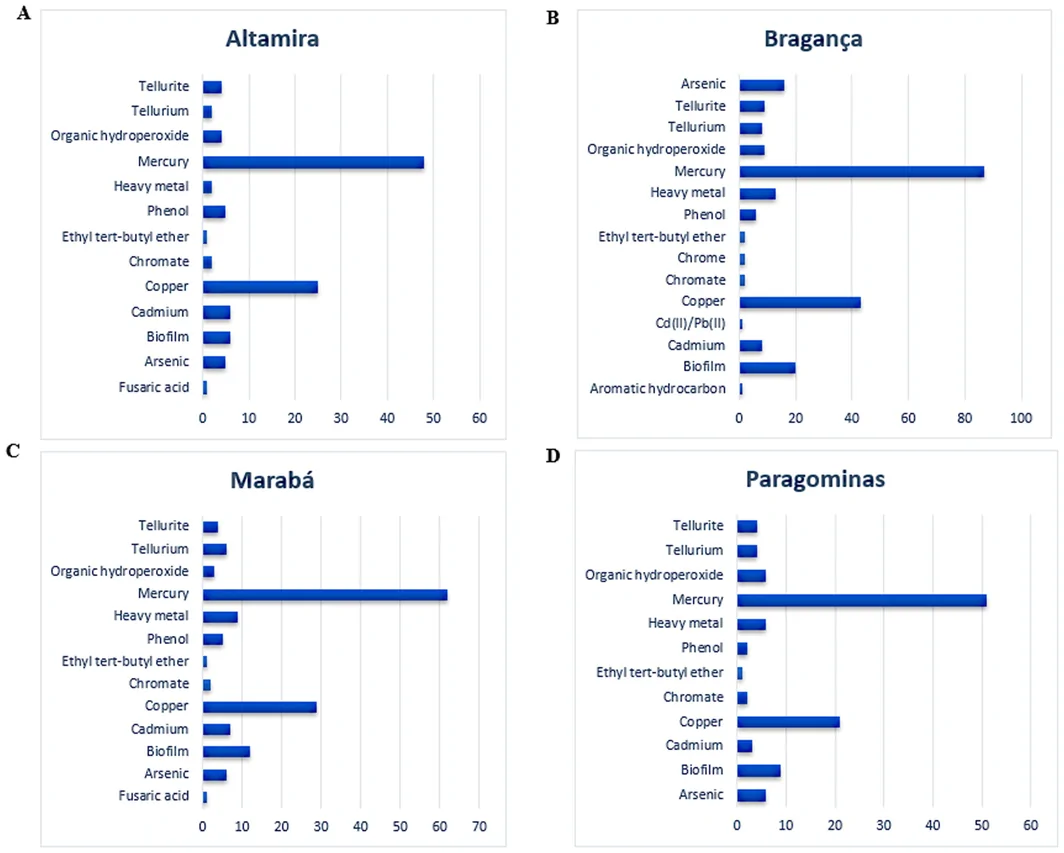
Distribution of bioremediation‐related genes by contaminant category in Altamira (A), Bragança (B), Marabá (C) and Paragominas (D). The plots show the number of genes assigned to each contaminant‐related category, highlighting the predominance of mercury‐ and copper‐associated functions across all municipalities.

The predominance of mercury‐ and copper‐related sequences may reflect both their representation in the sampled metagenomes and their disproportionate representation in the curated reference catalogue. Together, these two categories accounted for 62.27% of the catalogue, increasing the opportunity for metagenomic reads to be assigned to mercury‐ and copper‐associated entries. Therefore, their predominance should not be interpreted as direct evidence that mercury and copper were the most concentrated contaminants in the sampled waters. Instead, the observed pattern likely reflects the combined influence of metagenomic sequence representation and catalogue composition. Direct physicochemical measurements would be required to establish the environmental occurrence and concentration of these metals.

### 3.4 Microbial Diversity, Compositional Similarity and Statistical Support Among Sampling Sites

Pearson correlation heatmaps and alpha‐diversity indices were used to describe complementary aspects of the metagenomic profiles across Altamira, Bragança, Marabá and Paragominas (Figures 6, 7, 8, 9). Pearson correlation assessed similarity in relative gene‐abundance patterns among sampling sites, whereas the diversity indices characterized within‐site richness and evenness.

**FIGURE 6:**
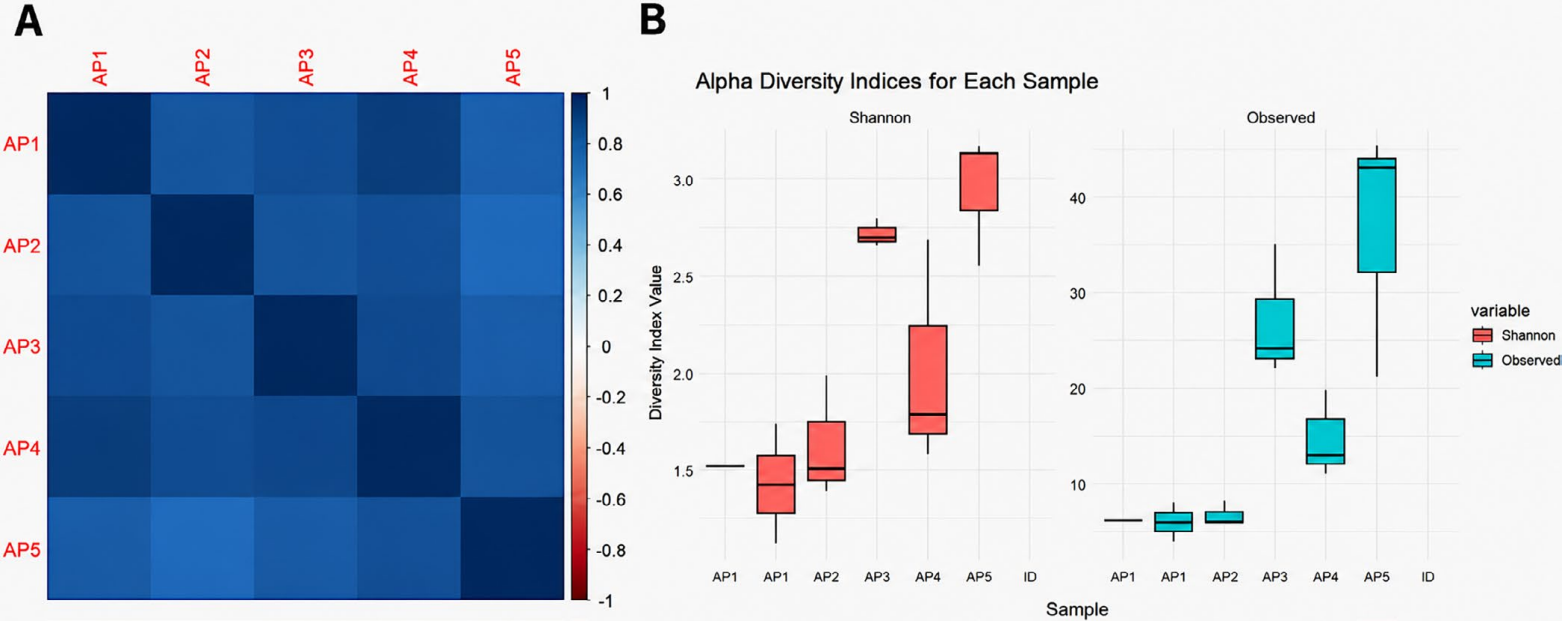
(A) Pearson correlation heatmap of sampling sites in Altamira based on metagenomic gene‐abundance profiles. Darker blue tones indicate stronger positive correlations and therefore greater similarity in relative abundance patterns across the evaluated genes. (B) Alpha‐diversity indices across the same sampling sites. The Shannon index represents functional gene diversity and evenness, whereas the Observed index represents detected gene richness.

**FIGURE 7:**
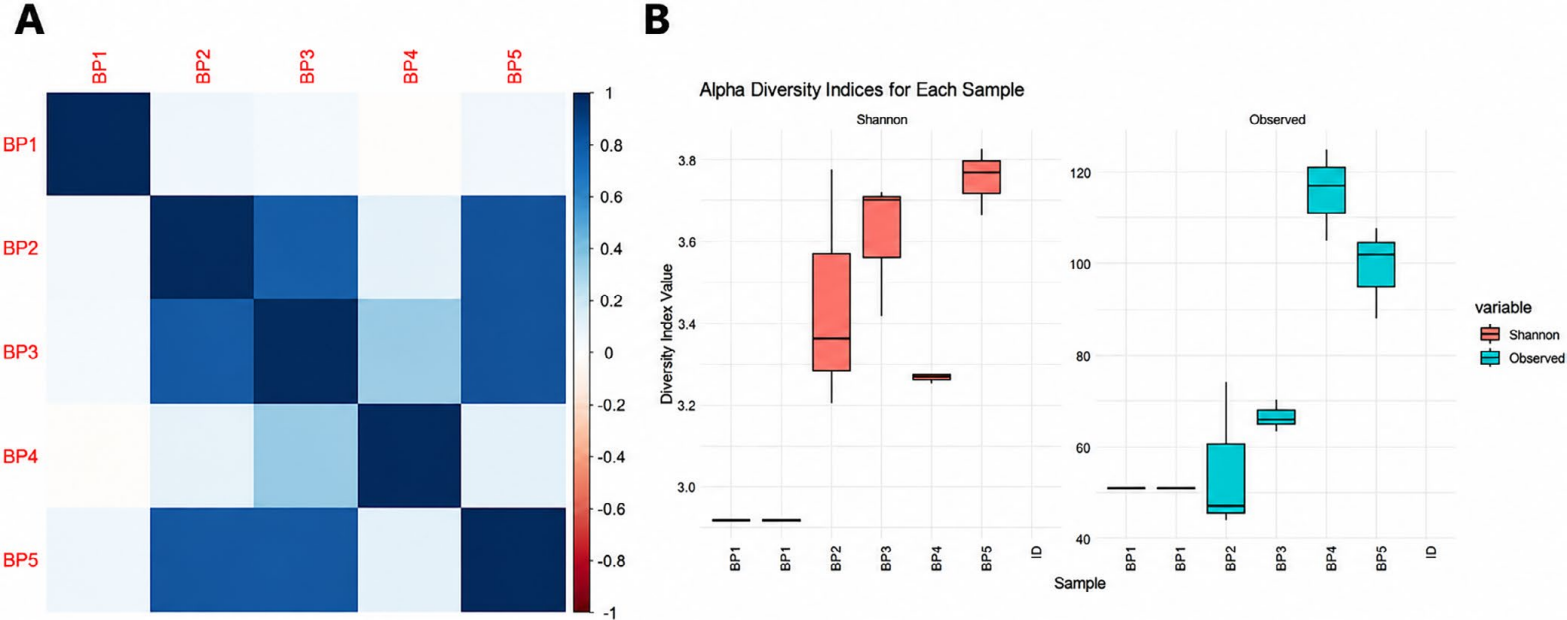
(A) Pearson correlation heatmap showing pairwise similarity among sampling sites in Bragança based on metagenomic gene‐abundance profiles. Darker blue tones represent stronger positive correlations, whereas lighter or reddish tones indicate weaker or negative correlations. (B) Alpha‐diversity indices across the same sampling sites. The Shannon index represents functional gene diversity and evenness, whereas the Observed index represents detected gene richness.

**FIGURE 8:**
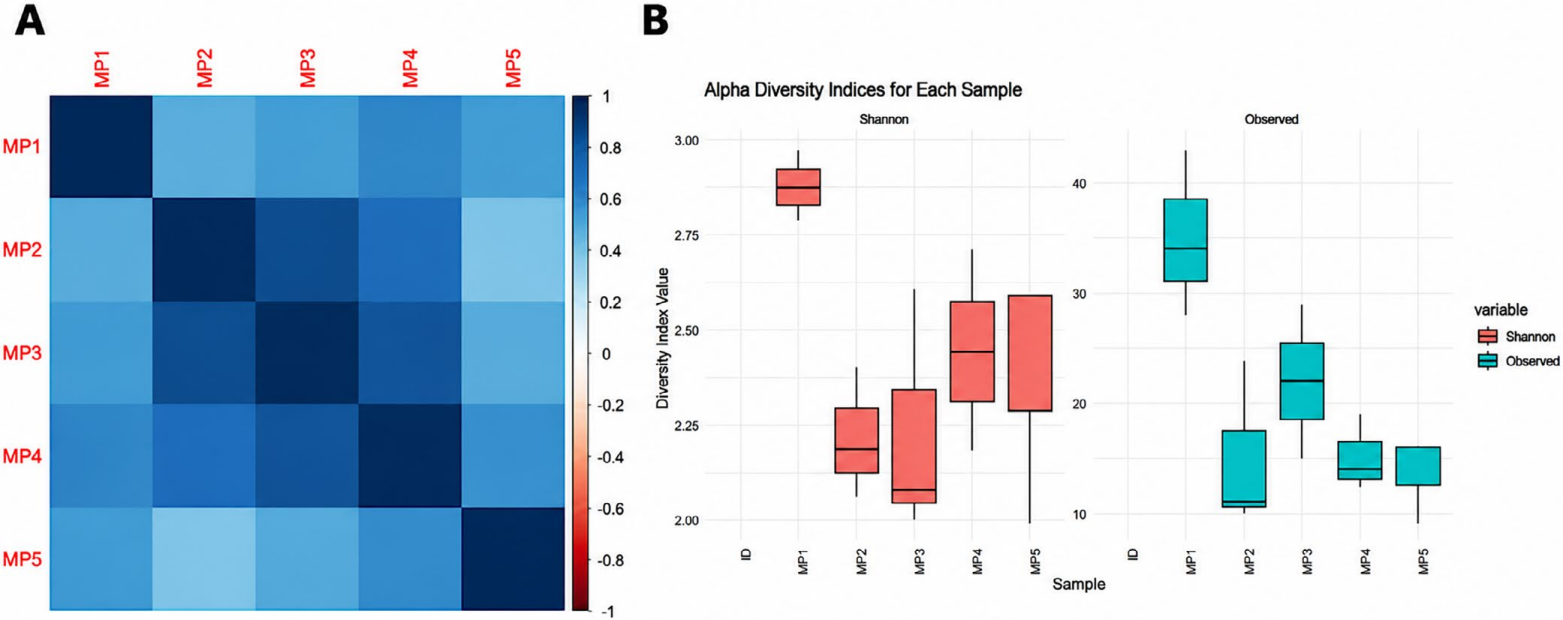
(A) Pearson correlation heatmap showing pairwise similarity among sampling sites in Marabá based on metagenomic gene‐abundance profiles. Darker blue tones represent stronger positive correlations, whereas lighter or reddish tones indicate weaker or negative correlations. (B) Alpha‐diversity indices across the same sampling sites. The Shannon index represents functional gene diversity and evenness, whereas the Observed index represents detected gene richness.

**FIGURE 9:**
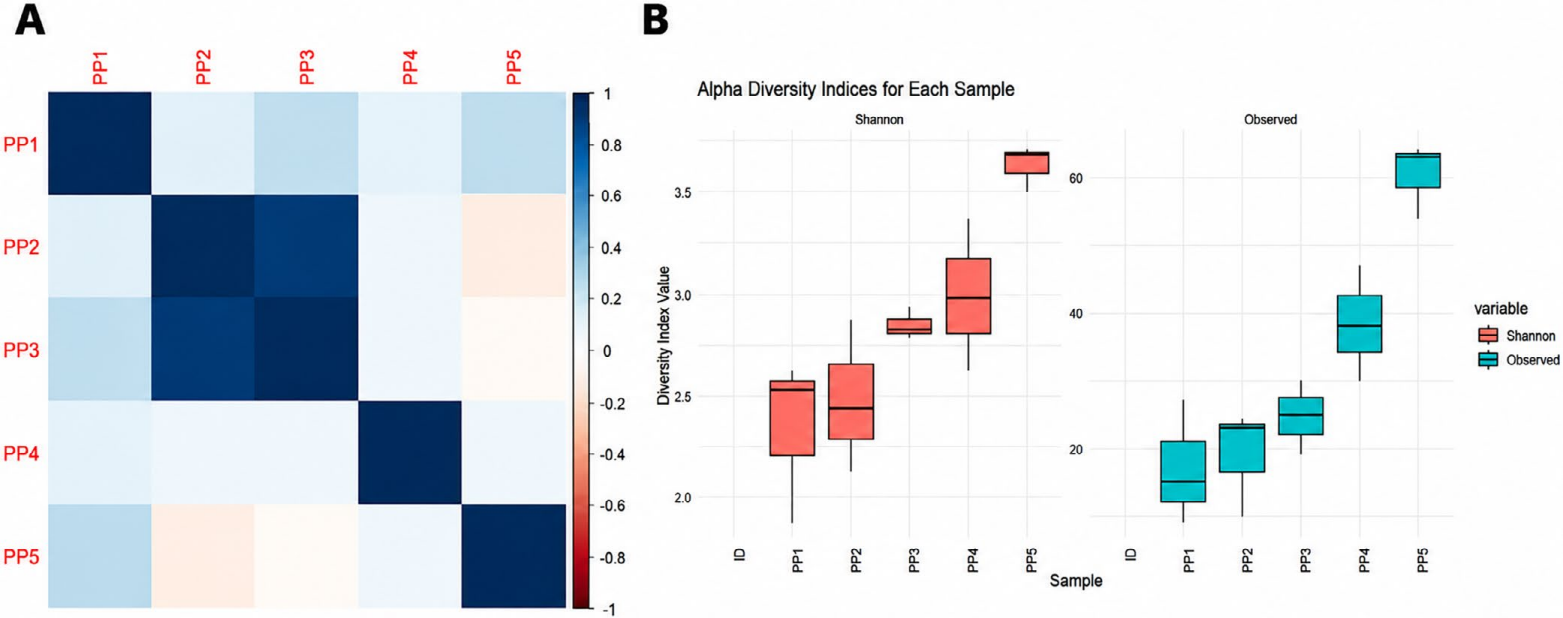
(A) Pearson correlation heatmap showing pairwise similarity among sampling sites in Paragominas based on metagenomic gene‐abundance profiles. Darker blue tones represent stronger positive correlations, whereas lighter or reddish tones indicate weaker or negative correlations. (B) Alpha‐diversity indices across the same sampling sites. The Shannon index represents functional gene diversity and evenness, whereas the Observed index represents detected gene richness.

Pearson correlation analysis revealed differences in the similarity of gene‐abundance profiles among sampling sites and municipalities. Altamira (Figure 6A) showed consistently high positive correlations among the five sites. In Bragança (Figure 7A), BP2 and BP3 displayed the strongest similarity, whereas BP1 showed lower correlations with the remaining sites. In Marabá (Figure 8A), MP2–MP4 exhibited highly correlated profiles, while MP1 was comparatively less similar to the other sampling points. In Paragominas (Figure 9A), stronger correlations occurred between PP2–PP3 and PP4–PP5, whereas PP1 and PP5 showed lower correlations with some of the remaining sites. These results describe similarity or divergence in relative abundance patterns and do not demonstrate equivalent environmental conditions, common contamination sources, or causal ecological relationships.

Functional diversity varied within and among the four river systems. In Altamira (Figure 6B), AP5 showed the highest Shannon diversity and observed gene richness, whereas AP1 and AP2 exhibited the lowest values. In Bragança (Figure 7B), BP5 displayed the highest functional diversity and richness, while BP3 showed the lowest. In Marabá (Figure 8B), MP1 had the highest diversity and gene richness, whereas MP2 and MP3 presented lower values. In Paragominas (Figure 9B), PP4 and PP5 displayed the highest diversity indices, while PP1 consistently showed the lowest values. These results indicate localized variation in the richness and evenness of the detected bioremediation‐related gene repertoire.

These results indicate similarity or divergence in the relative distribution of detected gene abundances, rather than equivalence in environmental conditions or contamination sources. Strong positive correlations suggest that pairs of sites exhibited comparable abundance patterns across the evaluated genes, even when their absolute abundances or gene compositions may have differed. Conversely, lower correlations identify sites with more distinct abundance profiles. Therefore, the Pearson analysis provides evidence of statistical similarity among functional profiles but does not establish causal ecological relationships or demonstrate exposure to the same contaminants. To complement the correlation and alpha diversity analyses, Jaccard distance matrices were used to assess compositional dissimilarity among biological replicates and sampling sites based on the presence/absence of bioremediation‐related genes (Figure 10A–D). This approach provided an additional perspective on spatial structuring by revealing how consistently functional profiles were maintained within each sampling point and how strongly they differed across the river gradients.

**FIGURE 10:**
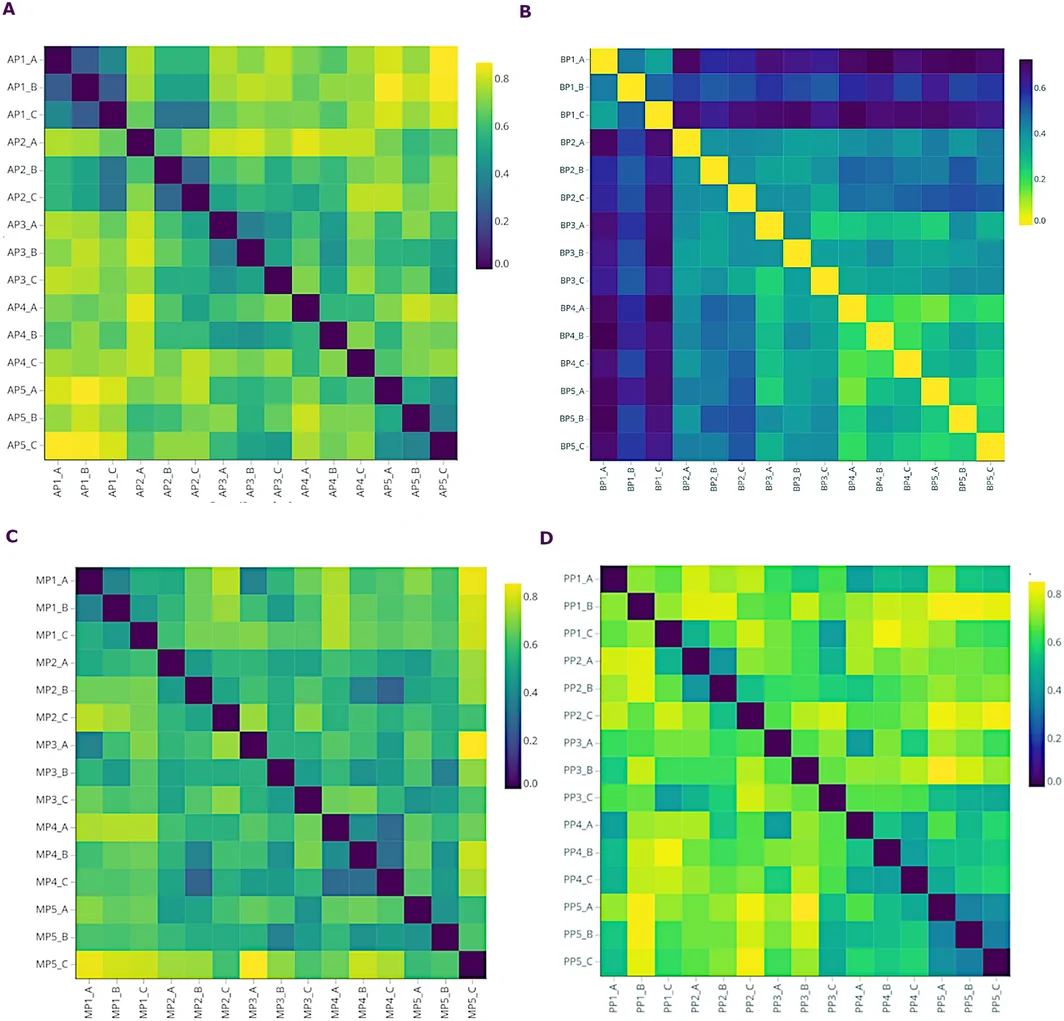
Jaccard distance heatmaps of metagenomic samples collected in Altamira (A) and Bragança (B), Pará, Brazil. Each matrix shows pairwise compositional dissimilarity among biological replicates and sampling sites based on the presence/absence of bioremediation‐related genes. Lower Jaccard distance values indicate greater similarity among samples, whereas higher values indicate greater compositional dissimilarity. The colour scale represents the magnitude of pairwise Jaccard distances, and the diagonal corresponds to self‐comparisons. Jaccard distance heatmaps for Marabá (C) and Paragominas (D), following the same analytical framework described for panels A–B.

Overall, the four municipalities showed moderate to high metagenomic heterogeneity, although the degree of local consistency varied among river systems (Tables [Link], [Link]). In Paragominas (Figure 10D), PP5 exhibited the highest within‐site consistency, indicating a relatively stable local metagenomic profile. In Altamira (Figure 10A), AP1 was the most compositionally consistent site, whereas AP2 displayed marked internal heterogeneity due to the divergence of one replicate from the remaining samples. In Bragança (Figure 10B), BP4 showed the highest within‐site similarity, while BP1 remained more distinct from the other sites and the downstream samples displayed greater compositional overlap. In contrast, Marabá (Figure 10C) exhibited the weakest geographic structuring and the highest overall heterogeneity, with relatively broad dissimilarity patterns among samples and limited spatial segregation along the river. Taken together, the Jaccard analysis identified both consistent within‐site profiles and substantial compositional variation among biological replicates and sampling points. Some sites maintained relatively similar presence–absence patterns among replicates, whereas others exhibited greater internal heterogeneity or overlap with neighbouring sites. These results indicate spatial variation in the composition of the detected gene repertoire but do not by themselves identify the environmental factors responsible for that variation.

In this study, a site of localized functional enrichment was defined descriptively as a sampling point showing one or more of the following characteristics: high relative gene abundance, recurrent pairwise differentiation, or a comparatively large site‐specific gene set. This designation does not indicate directly measured contaminant concentrations, confirmed microbial activity, or experimentally demonstrated remediation capacity.

Dunn's post hoc comparisons revealed distinct patterns of spatial differentiation among the four municipalities (Table 1). The main genes and pairwise contrasts are summarized in Table S5. Significant differences were concentrated at specific sites and varied according to the evaluated gene. In Altamira, significant contrasts were mainly concentrated at AP4–AP5, particularly for *TerC* and *Chrome_Resist*, indicating predominantly downstream differentiation. Bragança showed a more heterogeneous and gene‐dependent structure, with BP4–BP5 differing across markers such as *TerD*, *TehB* and *DUF446*. In Marabá, MP1 emerged as the most recurrently differentiated site, especially for *CutA1* and *DUF446*. Paragominas displayed the most consistent pattern, with PP5 repeatedly differing from the remaining sites for genes including *DUF386* and *TerD*. Overall, the Dunn analysis indicates that spatial structuring was present in all river systems, but varied from downstream or gene‐specific differentiation in Altamira and Bragança to more localized and recurrent patterns in Marabá and Paragominas.

**TABLE 1:** Summary of the main spatial differentiation patterns identified by Dunn's post hoc comparisons across the four municipalities.

Municipality	Recurrently differentiated site(s)	Representative genes	General pattern
Altamira	AP4–AP5	TerC, Chrome_Resist	Downstream differentiation
Bragança	BP4–BP5	TerD, TehB, DUF446	Gene‐dependent differentiation
Marabá	MP1	CutA1, DUF446	Recurrent localized differentiation
Paragominas	PP5	DUF386, TerD	Consistent differentiation

*Note:* The table presents the sampling sites that were recurrently differentiated, representative genes associated with significant pairwise contrasts, and the general spatial pattern observed in each river system.

Cross‐analysis convergence varied among municipalities because gene abundance, compositional consistency, pairwise differentiation and unique‐gene contribution represent distinct properties of the metagenomic profiles. MP1 showed the clearest convergence in Marabá, being highlighted by the abundance, Dunn and UpSet analyses, although the municipality exhibited considerable replicate heterogeneity. In Paragominas, PP5 was supported by gene‐abundance, Dunn and within‐site consistency patterns, whereas PP4 contained the largest site‐specific gene set. BP4 showed the strongest convergence in Bragança, while Altamira exhibited a broader downstream pattern involving AP3–AP5 rather than a single consistently differentiated site. Thus, the designation of localized enrichment was based on complementary evidence and does not imply direct measurement of contaminant concentration.

When interpreted together, Pearson correlation, Shannon diversity and Jaccard dissimilarity revealed complementary dimensions of spatial organization across the four river systems. Pearson correlations indicated that several sampling sites retained broadly similar abundance profiles, whereas Shannon diversity identified localized differences in richness and evenness, particularly at AP5, BP5, MP1 and PP4–PP5. Jaccard dissimilarity further showed that similarity in overall abundance patterns did not necessarily imply identical gene composition, since some sites and biological replicates exhibited substantial presence–absence turnover. Thus, the combined analyses suggest that the studied rivers share a broad functional background while retaining localized differences in diversity, composition and replicate consistency. These patterns should be interpreted as spatial associations rather than direct evidence of contaminant‐driven selection, because environmental contaminant concentrations were not measured directly.

### 3.5 Shared and Site‐Specific Bioremediation Genes

The UpSet diagrams for Altamira, Bragança, Marabá and Paragominas (Figures 11A,B and 12A,B) show how bioremediation‐related genes were distributed and shared among sampling sites. Across the four river systems, one or two sites generally contained the largest total or site‐specific gene sets, whereas a smaller group of genes was shared by all five sampling points within each municipality.

**FIGURE 11:**
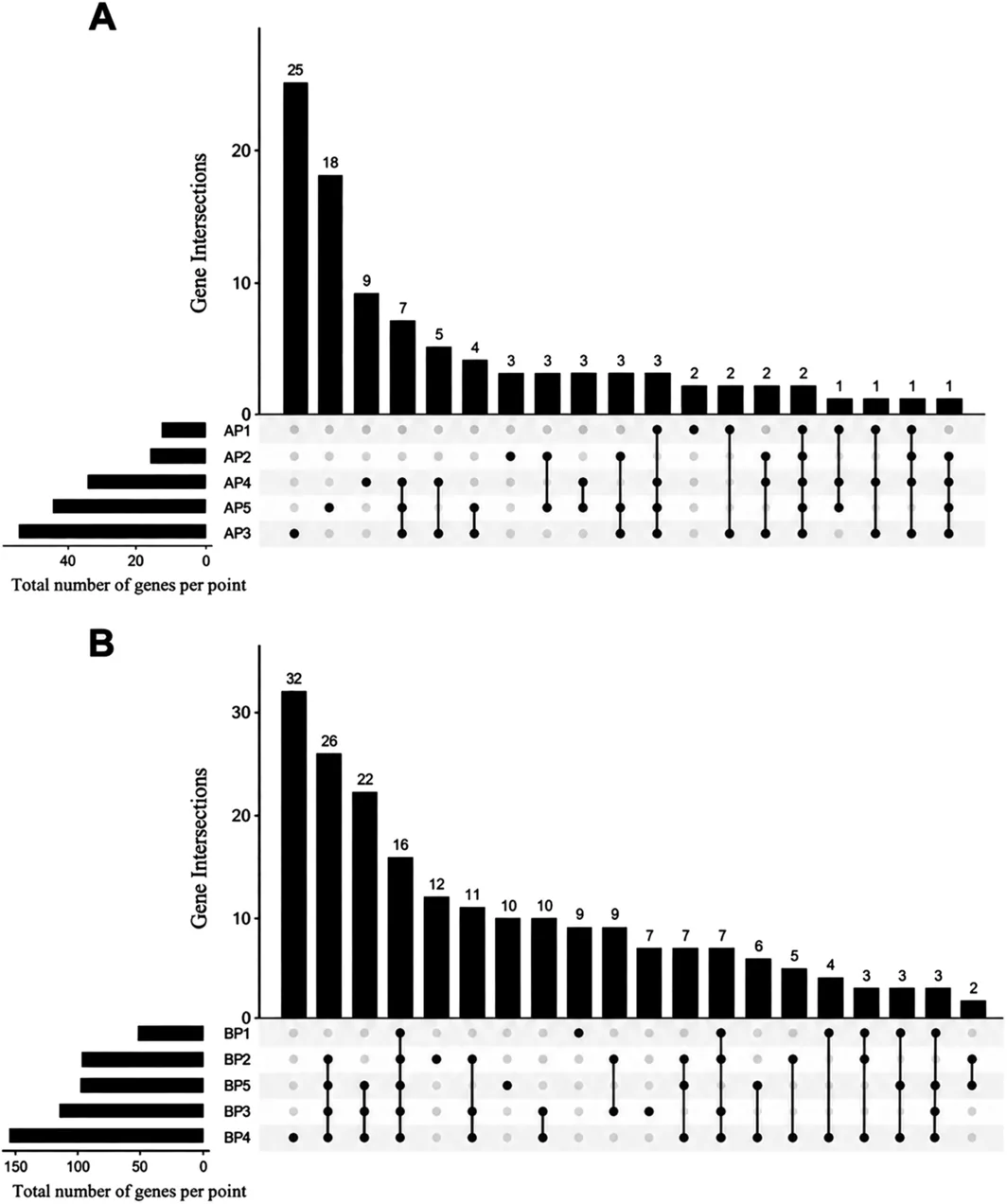
Shared and site‐specific bioremediation genes across sampling sites in Altamira (A) and Bragança (B). UpSet plots summarizing the distribution and overlap of bioremediation‐related genes among the five sampling points in each river system (AP1–AP5 in Altamira and BP1–BP5 in Bragança). Horizontal bars indicate the total number of genes detected at each site. The matrix of filled circles and connecting lines represents all combinations of sites (intersections), and the corresponding vertical bars show the number of genes present exclusively in each combination. Single‐site intersections correspond to genes unique to a given sampling point, whereas multi‐site intersections represent genes shared by two or more points. These diagrams highlight both a small core resistome shared within each river and a substantial fraction of genes restricted to one or a few sites.

**FIGURE 12:**
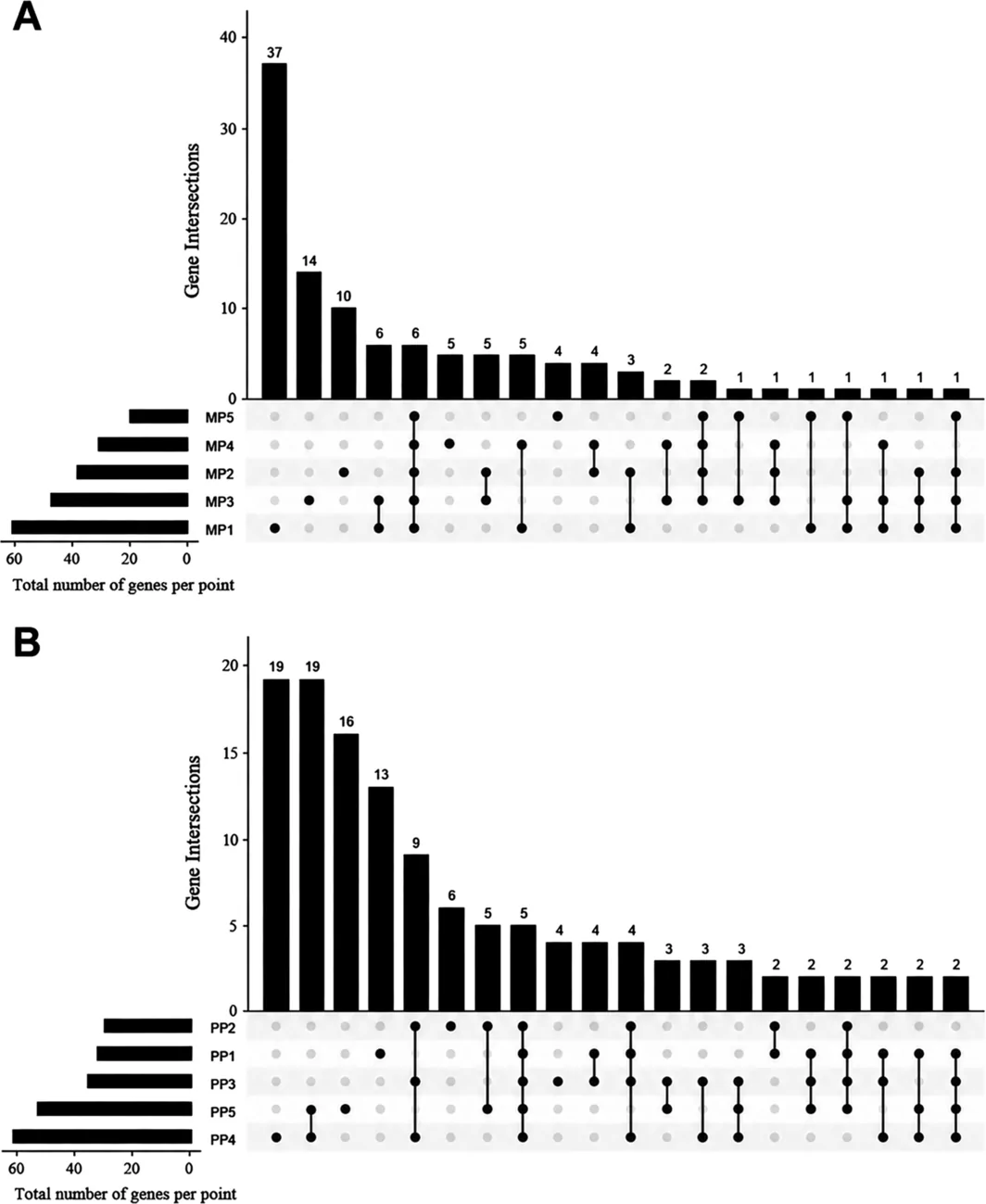
Shared and site‐specific bioremediation genes across sampling sites in Marabá (A) and Paragominas (B). UpSet plots summarizing the distribution and overlap of bioremediation‐related genes among the five sampling points along the Itacaiúnas River (MP1–MP5) and the Uraim River (PP1–PP5). Horizontal bars represent the total number of genes detected at each site. The matrix of filled circles and connecting lines indicates the different combinations of sites, and the corresponding vertical bars show the number of genes in each intersection (shared or exclusive). Single‐site intersections indicate genes unique to a given sampling point, while combinations of sites represent genes shared by those points only. Together, these plots reveal a mixture of core genes common to most sites and highly localized gene sets characterizing sites of localized functional enrichment within each river system.

UpSet analyses revealed a combination of shared and site‐specific bioremediation‐related genes across the four river systems (Table 2). Bragança showed the largest overall gene repertoire, with 213 genes and a core of 16 genes shared by all five sites. Marabá displayed the greatest site‐specific contribution, with 37 genes restricted to MP1. Altamira and Paragominas showed smaller core components, comprising two and five genes, respectively, while AP3 and PP4 contained the largest unique gene sets within these municipalities.

**TABLE 2:** Summary of total, core and site‐specific bioremediation‐related genes identified across the four municipalities based on the UpSet analyses.

Municipality	Total genes	Core genes	Site with most unique genes	Unique genes
Altamira	95	2	AP3	25
Bragança	213	16	BP4	32
Marabá	111	6	MP1	37
Paragominas	131	5	PP4	19

*Note: Total genes* represent the total number of genes detected in each municipality, whereas *core genes* correspond to genes shared by all five sampling sites. *Site with* the *most unique genes* indicates the sampling point with the largest single‐site intersection, and *unique genes* represent the number of genes detected exclusively at that site.

Together, these results indicate that each river system contained a limited common functional component combined with substantial localized variation. Sites containing comparatively large unique gene sets may represent relevant targets for future bioprospecting and functional validation. However, their designation as sites of localized enrichment reflects the distribution of detected sequences and should not be interpreted as evidence of greater contaminant concentration or confirmed microbial remediation activity.

### 3.6 Key Microbial Taxa and Bioprospecting Implications

Across the four municipalities, a recurrent group of bacterial taxa was associated, through metagenomic inference, with the detected bioremediation‐related genes. The main sites of localized enrichment, predominant contaminant‐related categories, and recurrent taxa are summarized in Table 3. *Acinetobacter baumannii* , *Enterobacter ludwigii* , *Pseudomonas aeruginosa* , *Stenotrophomonas maltophilia* and *Cupriavidus metallidurans* were among the most frequently detected taxa, particularly at AP3–AP5, BP4–BP5, MP1 and PP4–PP5. These taxa were mainly associated with mercury‐ and copper‐resistance functions, although genes related to arsenic transformation, cadmium/lead resistance, tellurium tolerance, phenol degradation, organic hydroperoxide resistance and biofilm formation were also detected. Together, these patterns identify a limited set of recurrent taxa associated with both shared and localized components of the resistome across the four river systems.

**TABLE 3:** Summary of the main sites showing localized enrichment of bioremediation‐related genes, the predominant contaminant‐related categories, and the recurrent bacterial taxa inferred from metagenomic data in each municipality.

Municipality	Main enriched site(s)	Main contaminant‐related categories	Recurrent inferred taxa
Altamira	AP3–AP5	Mercury, copper	*Acinetobacter baumannii* , *Cupriavidus metallidurans*
Bragança	BP4–BP5	Mercury, copper	*Acinetobacter baumannii* , *Pseudomonas aeruginosa*
Marabá	MP1	Mercury, copper	*Enterobacter ludwigii* , *Stenotrophomonas maltophilia*
Paragominas	PP4–PP5	Mercury, copper	*Acinetobacter baumannii* , *Enterobacter ludwigii*

*Note:* Taxonomic assignments indicate associations with the detected resistome and do not represent experimentally isolated or functionally validated microorganisms.

These associations should be interpreted as indicators of functional potential rather than evidence of confirmed remediation activity. Metagenomic detection does not demonstrate gene expression, microbial viability, pollutant removal efficiency, or the direct contribution of individual taxa to environmental remediation. Experimental isolation, cultivation and functional validation are therefore required before these microorganisms can be considered for biotechnological applications. Moreover, the recurrent detection of opportunistic or potentially pathogenic taxa, particularly *A. baumannii* and *P. aeruginosa* , highlights the need for careful biosafety and ecological assessment when selecting candidates for future bioprospecting studies.

## 4 Conclusion

This study shows that Amazonian aquatic ecosystems harbour a spatially structured repertoire of bioremediation‐related genes, with strong representation of mercury‐ and copper‐associated functions. By combining a curated catalogue with metagenomic screening, we identified both shared resistome components and site‐specific hotspots across four river systems in Pará. These results support the value of the curated catalogue as a useful genomic reference for molecular screening and environmental surveillance. However, the study is based on curated reference data and metagenomic inference and therefore does not by itself demonstrate actual remediation performance. Future studies should focus on experimental validation, microbial isolation and functional testing of the most relevant candidates.

## Author Contributions

**Thais Mayara Castro dos Anjos:** writing – original draft, methodology, writing – review and editing, data curation, conceptualization. **Daralyns Borges Macedo:** data curation. **Victor Benedito Costa Ferreira:** software, methodology. **Carlos Willian Dias Dantas:** software, methodology. **Edian Franklin Franco de Los Santos:** funding acquisition, methodology. **Oscar Victor Cardenas‐Alegria:** methodology, writing – review and editing. **Rommel Thiago Jucá Ramos:** conceptualization, funding acquisition, writing – review and editing, supervision.

## Funding

This work was supported by Pró‐Reitoria de Pesquisa e Pós‐Graduação, Universidade Federal do Pará, Coordenação de Aperfeiçoamento de Pessoal de Nível Superior, Conselho Nacional de Desenvolvimento Científico e Tecnológico, 312316/2022‐4 and Secretaria de Estado de Ciência, Tecnologia, Educação Superior, Profissional e Tecnológica do Pará.

## Conflicts of Interest

The authors declare no conflicts of interest.

## Supporting information


**Figure S1:** Hierarchical heatmap of bioremediation‐related genes detected across the sampling sites in Altamira (AP1–AP5). Rows correspond to reference genes and columns to sampling sites. Colours represent gene‐wise standardized abundance values (z‐scores), ranging from lower (blue) to higher (red) relative abundance.

**Figure S2:** Hierarchical heatmap of bioremediation‐related genes detected across the sampling sites in Bragança (BP1–BP5). Rows correspond to reference genes and columns to sampling sites. Colours represent gene‐wise standardized abundance values (z‐scores), ranging from lower (blue) to higher (red) relative abundance.

**Figure S3:** Hierarchical heatmap of bioremediation‐related genes detected across the sampling sites in Marabá (MP1–MP5). Rows correspond to reference genes and columns to sampling sites. Colours represent gene‐wise standardized abundance values (z‐scores), ranging from lower (blue) to higher (red) relative abundance.

**Figure S4:** Hierarchical heatmap of bioremediation‐related genes detected across the sampling sites in Paragominas (PP1–PP5). Rows correspond to reference genes and columns to sampling sites. Colours represent gene‐wise standardized abundance values (z‐scores), ranging from lower (blue) to higher (red) relative abundance.

**Table S1:** Pairwise Jaccard distance matrix among metagenomic samples collected in Altamira, Pará, Brazil. Rows and columns correspond to biological replicates from sampling sites AP1–AP5. Values indicate pairwise compositional dissimilarity based on the presence/absence of bioremediation‐related genes.

**Table S2:** Pairwise Jaccard distance matrix among metagenomic samples collected in Bragança, Pará, Brazil. Rows and columns correspond to biological replicates from sampling sites BP1–BP5. Values indicate pairwise compositional dissimilarity based on the presence/absence of bioremediation‐related genes.

**Table S3:** Pairwise Jaccard distance matrix among metagenomic samples collected in Marabá, Pará, Brazil. Rows and columns correspond to biological replicates from sampling sites MP1–MP5. Values indicate pairwise compositional dissimilarity based on the presence/absence of bioremediation‐related genes.

**Table S4:** Pairwise Jaccard distance matrix among metagenomic samples collected in Paragominas, Pará, Brazil. Rows and columns correspond to biological replicates from sampling sites PP1–PP5. Values indicate pairwise compositional dissimilarity based on the presence/absence of bioremediation‐related genes.

**Table S5:** Detailed results of Dunn's post hoc pairwise comparisons among sampling sites in the four municipalities. The table presents the bioremediation‐related genes showing significant pairwise differences among sampling sites in Altamira, Bragança, Marabá and Paragominas, together with the corresponding comparison pairs and adjusted *p*‐values. Dunn's test was performed following the Kruskal–Wallis analysis, with *p*‐values adjusted using the Bonferroni correction. Comparisons with adjusted *p* < 0.05 were considered statistically significant.

## Data Availability

The curated bioremediation gene catalogue used in this study is publicly available in the following GitHub repository: https://github.com/labgm/Bioprospecting‐of‐Bioremediating‐Microorganisms‐in‐Amazonian‐Aquatic‐Ecosystems. The repository contains the nucleotide and protein sequence files corresponding to the curated version of the database used in the present manuscript. Because this resource is still undergoing curation, expansion and refinement, the deposited version should be regarded as the version associated with the present study, whereas a more comprehensive and fully documented release is planned for a future dedicated publication.
